# The impact of pain neuroscience education on physical therapy students’ pain knowledge, attitudes, beliefs, and clinical recommendations: a randomized controlled trial

**DOI:** 10.1186/s12909-026-09994-y

**Published:** 2026-07-27

**Authors:** Ahmad Muhsen, Bodor Bin Sheeha

**Affiliations:** 1 Department of Physical Therapy, Faculty of Applied Medical Sciences, The Hashemite University, P.O box 330127, Zarqa, 13133 Jordan; 2 Department of Rehabilitation Sciences, College of Health and Rehabilitation Sciences, Princess Nourah Bint Abdulrahman University, Riyadh, Saudi Arabia

**Keywords:** Pain neuroscience education, Physiotherapy education, Undergraduate physiotherapy students, Pain knowledge, Pain-related attitudes, Guideline-concordant care, Biopsychosocial model

## Abstract

**Background:**

Pain neuroscience education (PNE) promotes a biopsychosocial understanding of pain among health-professional students, yet evidence for its long-term effects outside high-income countries remains limited. This study evaluated the immediate and six-month effects of a single PNE lecture on pain knowledge, attitudes, beliefs, and clinical recommendations in Jordanian physiotherapy students.

**Methods:**

A parallel-group randomized controlled trial was conducted with first- and second-year physiotherapy students. Students were randomized (1:1; 60 per group) to a 70-minute PNE lecture or an attention-matched musculoskeletal red flags lecture. Primary outcomes were pain knowledge (Revised Neurophysiology of Pain Questionnaire [RNPQ]) and pain-related attitudes (modified Health Care Providers’ Pain and Impairment Relationship Scale [HC-PAIRS]). The secondary outcome was guideline-concordant recommendations assessed using a validated vignette. Analyses included linear and logistic mixed-effects models, with responder analyses using minimal clinically important difference thresholds to estimate numbers needed to treat (NNTs).

**Results:**

All students (*N* = 120) completed the study with no attrition. Significant group × time interactions were observed for pain knowledge and attitudes (both *p* < 0.001). Post-intervention, the PNE group demonstrated higher pain knowledge scores than the control group (adjusted mean difference = 2.46, 95% CI 1.68–3.24) and more favorable attitudes (adjusted mean difference = − 7.54, 95% CI − 10.62 to − 4.46). At six months, knowledge gains remained statistically significant (1.20, 95% CI 0.25–2.15; *p* = 0.020), while the between-group difference for attitudes was no longer statistically significant (–3.60, 95% CI − 7.25 to 0.05; *p* = 0.060). Guideline-concordant clinical recommendations improved immediately after PNE (adjusted ORs 2.95–6.18) but were not sustained at follow-up (all *p* > 0.05). Responder analyses demonstrated substantial immediate educational benefit (NNT 2.7 for knowledge, 3.6 for attitudes), which decreased at six months, consistent with attenuation of intervention effects over time.

**Conclusion:**

A single 70-minute PNE session produced significant and clinically meaningful immediate improvements in pain knowledge, attitudes, and guideline-concordant clinical recommendations. At six months, pain knowledge remained significantly improved, whereas the remaining effects attenuated. These findings support consideration of embedding PNE early within pre-licensure physiotherapy curricula. Future research should determine whether recursive reinforcement improves longer-term retention of educational gains.

**Trial registration:**

The study protocol was prospectively registered at ClinicalTrials.gov (NCT06821022) on February 6, 2025.

**Supplementary Information:**

The online version contains supplementary material available at https://doi.org/10.1186/s12909-026-09994-y.

## Introduction

Chronic musculoskeletal pain conditions are among the most prevalent contributors to global disability, increased healthcare utilization, and impaired health-related quality of life [1–4]. Modern clinical low back pain (LBP) guidelines increasingly advocate a biopsychosocial approach to care that considers physical, psychological, and social factors rather than relying solely on tissue-based explanations [5]. Despite this shift, maladaptive biomedical pain beliefs remain common among healthcare professionals and students, contributing to under-recognition of psychosocial factors and poorer patient outcomes [6, 7].

Pain-related attitudes and beliefs among healthcare providers are associated with clinical recommendations and recognition of psychosocial factors, highlighting early professional education as a critical target for intervention [8, 9]. Evidence consistently demonstrates that undergraduate pain education remains substantially underemphasized. Briggs et al. [10] described undergraduate pain education as “woefully inadequate,” with less than 1% of total curriculum time dedicated to pain. Similarly, subsequent international reports have demonstrated limited adherence to the International Association for the Study of Pain (IASP) pre-licensure guidelines [11]. This educational deficit is concerning because growing evidence links inadequate pain education to the persistence of negative beliefs and suboptimal management of pain [12].

Pain neuroscience education (PNE) has emerged as an evidence-based educational approach to improve conceptual understanding of pain. PNE aims to reconceptualize pain as a protective yet modifiable output of a sensitive nervous system and a multidimensional response to perceived threat, influenced by contextual and cognitive factors [13]. Although originally developed for patients, PNE has been increasingly adapted for healthcare students. Randomized controlled trials (RCTs) and longitudinal studies have demonstrated meaningful improvements in pain knowledge, attitudes, and clinical recommendations immediately following single 70-minute PNE sessions among physiotherapy and sports-rehabilitation undergraduates [14–16]. However, key evidence gaps persist regarding long-term retention of educational gains, translation into guideline-concordant clinical recommendations using validated measures, and generalizability of findings beyond Western, high-income contexts.

Regional and culturally proximate evidence from the Middle East and neighboring contexts suggests that PNE may be effective, despite significant heterogeneity in the durability of educational outcomes. In Turkey, a 70-minute PNE session produced substantial immediate gains in pain knowledge with partial retention at six months, though attitudinal change was modest [16]. In Saudi Arabia, a 21-hour PNE program improved pain knowledge among physiotherapists without significantly changing pain-related attitudes [17]. Recent evidence from Jordan has demonstrated limited pain knowledge and persistent biomedical beliefs among final-year physiotherapy and occupational therapy students, further highlighting the need to strengthen pain education locally [18]. Collectively, these findings reflect the context-dependent nature of PNE implementation and the importance of region-specific evaluations.

To our knowledge, no randomized controlled trial has evaluated PNE among undergraduate physiotherapy students in Jordan, and evidence from Arab Middle Eastern pre-licensure physiotherapy programs remains limited. This study aimed to evaluate the effectiveness of a single 70-minute PNE lecture on improving pain neurophysiology knowledge, pain-related attitudes and beliefs, and guideline-concordant clinical recommendations among undergraduate physiotherapy students.

## Methods

### Design

A parallel-group randomized controlled trial (RCT) was conducted at the Faculty of Applied Medical Sciences, The Hashemite University, Jordan. Students were individually randomized in a 1:1 ratio to receive either a pain neuroscience education (PNE) lecture or an attention-matched lecture on musculoskeletal red flags. Both sessions were delivered in a group classroom format, standardized to 70 min. Outcomes were measured at baseline, immediately post-intervention and at six-month follow-up.

### Randomization, masking, and contamination control

An independent research assistant (RA), who was not involved in recruitment, teaching, outcome assessment, or data analysis, generated the allocation sequence using a computer-based random number generator. After providing written informed consent and completing baseline assessments as a single cohort, participants were randomly allocated to Group A or Group B according to a concealed allocation list solely held by the RA.

Because both lectures were delivered by a single educator, sessions were held sequentially on the same day, and groups were kept separate before and after their assigned sessions to minimize crossover and contamination. Students allocated to Group A remained in the lecture hall for the first session, whereas students assigned to Group B exited the hall and returned later for the control lecture. To prevent cross-group interaction, group transitions and entry/exit were supervised by the RA. The RA verified attendance at each session against the randomization list to ensure adherence to group assignment and prevent crossover.

Session labels (“Group A” and “Group B”) were used to maintain neutral framing. The educator was unaware of which allocation label corresponded to each educational condition until immediately before delivery. Students were informed that the study compared two forms of pain-related education but were not told which session was hypothesized to be superior. Because participant masking to educational content was not feasible, expectancy equivalence, hypothesis awareness, and potential contamination were assessed after the intervention, consistent with methodological recommendations for trials in which full content masking is difficult or incomplete [19, 20].

Immediately after the intervention, participants completed adapted credibility and expectancy items based on the Credibility and Expectancy Questionnaire (CEQ) [21], along with study-specific items assessing allocation guess, hypothesis awareness, and contamination (Additional file 1: Table S1). These items were used descriptively to evaluate credibility, expectancy, masking integrity, and potential contamination. Potential cross-group discussion was evaluated immediately post-session and again at six-month follow-up. Statistical analyses were conducted using coded allocation labels (0 = control, 1 = PNE), with group identity concealed until completion of the primary analyses.

### Participants

Undergraduate physiotherapy students enrolled in their first or second year of study at The Hashemite University (Jordan) were eligible to participate. Inclusion criteria were active enrolment in the first or second year of study and provision of informed consent. Exclusion criteria were prior participation in formal pain neuroscience education training, involvement in the development or validation of the study vignette instrument, and enrolment beyond the second year of the program. Recruitment was conducted through email announcements, campus advertisements, and brief five-minute in-class presentations. All participants provided written informed consent prior to enrolment. Students were informed that participation was voluntary and that non-participation or withdrawal would not affect their academic standing or grades. Targeting students at this formative stage was intended to evaluate PNE before substantial exposure to advanced pain-related coursework, consistent with previous student-focused PNE trials [14–16, 22].

The study protocol was approved by the Institutional Review Board (IRB) of The Hashemite University (HU-IRB No. 33/1/2024/2025) and implemented in accordance with the ethical principles of the Declaration of Helsinki. Data were collected between May and December 2025. This trial was prospectively registered with *ClinicalTrials.gov* (identifier: NCT06821022). To minimize contamination, students were asked to avoid discussing lecture content or questionnaire details with peers and, where possible, to avoid seeking additional educational materials on pain neuroscience during the active study period.

### Procedure

At the start of the study, all students provided written informed consent and completed baseline measures as a single cohort. Following randomization, they attended their assigned educational session (PNE or red-flags education). Immediately after the session, students completed post-intervention assessments, including the primary outcome measures and adapted credibility/expectancy and masking-assessment items. Follow-up assessments were conducted six months later. To maximize retention and minimize attrition, the six-month follow-up assessments were scheduled during regular timetabled teaching activities, with advance reminders provided through course announcements and email communication. Participation in research assessments remained voluntary throughout the study.

After completion of all follow-up assessments, participants attended a short debriefing session led by the primary investigator. The session clarified the study’s purpose and the rationale for the two educational approaches, and addressed participant questions.

**Fig. 1:**
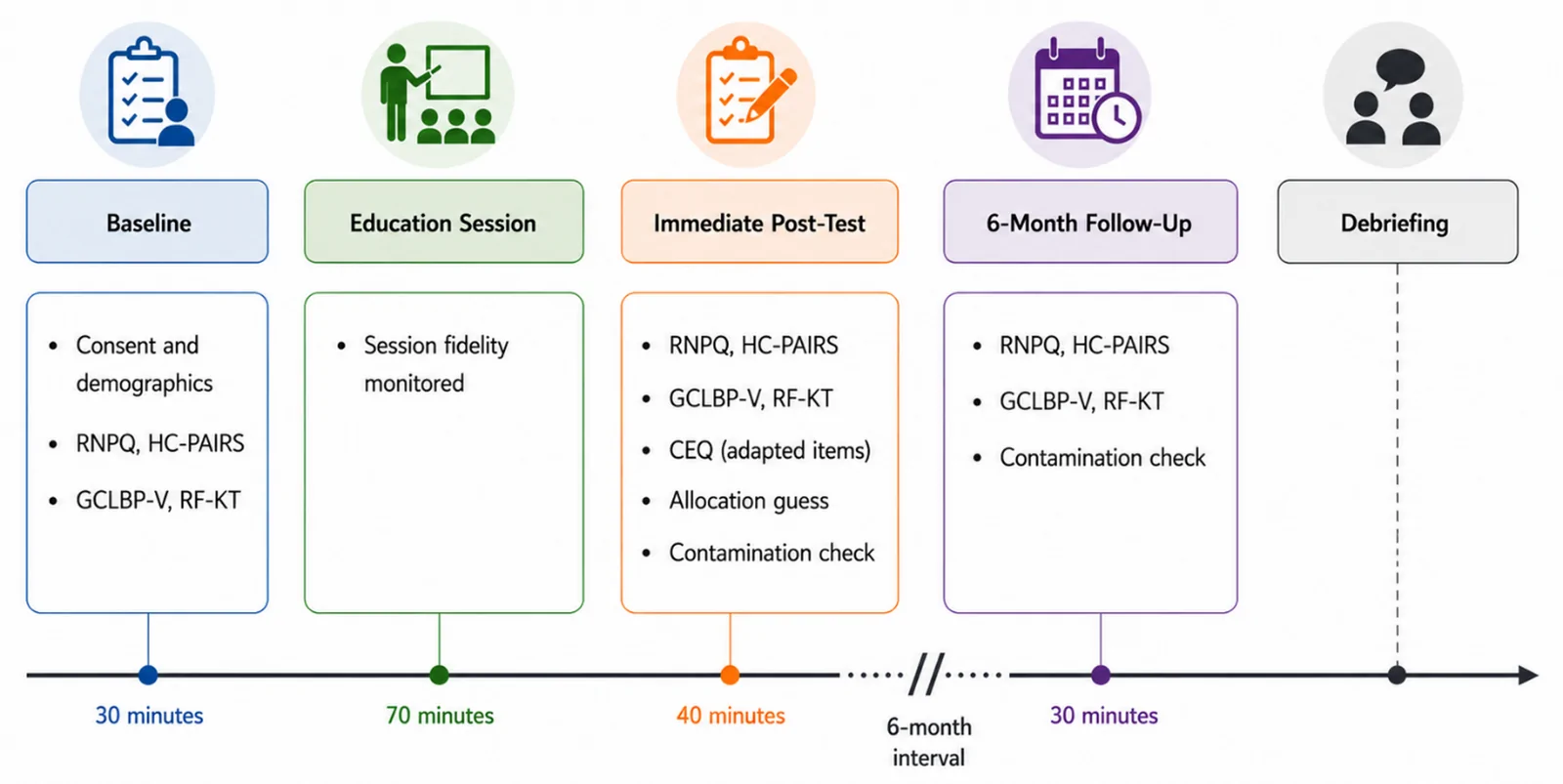
Study procedure and assessment timeline. RNPQ, Revised Neurophysiology of Pain Questionnaire; HC-PAIRS, Health Care Providers’ Pain and Impairment Relationship Scale; GCLBP-V, Guideline-Concordant Low Back Pain Vignette; RF-KT, Red Flags Knowledge Test; CEQ, Credibility and Expectancy Questionnaire

The overall sequence of study activities, including assessment points and follow-up timing, is illustrated in Fig. 1.

### Interventions

A single physiotherapist pain educator (AM), with extensive clinical experience and formal training in pain neuroscience education (PNE), delivered both educational sessions. Each session lasted 70 min, delivered in a group classroom format using PowerPoint slides and a standardized teaching script. To ensure attention matching, the sessions were equal in duration, format, and structure. Each intervention was delivered to a separate student cohort to prevent cross-contamination, and both concluded with a brief question-and-answer period, when time permitted.

### Pain Neuroscience Education (PNE) session

The PNE session was based on the *Explain Pain* model [13]. Core content included pain neurophysiology concepts such as: nociceptive transmission, peripheral and central sensitization, descending pain modulation, and the influence of biopsychosocial (psychological and contextual) factors on pain perception. The session also incorporated metaphors, drawings, and brief case scenarios to illustrate key concepts. The primary goal was to reconceptualize pain as a protective yet potentially maladaptive neurophysiological output modulated by biopsychosocial factors.

### Red-Flags education session (attention control)

The control session focused exclusively on red-flag screening for low back pain (LBP). The content was derived from established international clinical guidelines and systematic reviews [5, 23–29] and focused on features of serious or sinister pathology (e.g. malignancy, infection, vertebral fracture, and cauda equina syndrome). The lecture covered history-taking, systemic signs, and referral decision-making, without inclusion of any pain neurophysiology or biopsychosocial material.

### Intervention fidelity and contamination control

Intervention fidelity was formally monitored during experimental sessions. All teaching materials were finalized before the study and remained unaltered. The educator’s adherence to the experimental protocol was independently audited through in-person observation by a physiotherapist experienced in pain education, using a pre-defined fidelity checklist [30] (Additional file 1: Appendix S1). Adherence was defined a priori as ≥ 90% delivery of planned content with no inclusion of materials from the other condition. Instructor neutrality in tone and delivery was reinforced through specific training and rehearsal.

Between-group contamination was minimized by using a single educator to standardize delivery, using independently developed educational content to minimize conceptual overlap, scheduling separate cohorts sequentially to prevent cross-talk, and neutrally framing the study purpose.

### Study hypotheses

Based on the prospectively registered study objectives and predefined outcomes (ClinicalTrials.gov Identifier: NCT06821022), we hypothesized that students receiving pain neuroscience education (PNE) would demonstrate greater improvements in pain neurophysiology knowledge and more favorable biopsychosocial pain-related attitudes than those receiving the attention-control intervention immediately after the educational session. We further hypothesized that these improvements would persist at six-month follow-up. Secondary analyses examined whether PNE improved guideline-concordant clinical recommendations. The reported outcomes and assessment time points were consistent with the registered trial protocol.

### Outcome measures

The predefined primary outcomes were pain neurophysiology knowledge, assessed using the Revised Neurophysiology of Pain Questionnaire (RNPQ) [31], and pain-related attitudes and beliefs, assessed using the modified Health Care Providers’ Pain and Impairment Relationship Scale (HC-PAIRS) [32]. These outcomes were designated as primary because they represent the principal educational targets of pain neuroscience education. The secondary outcome was guideline-concordant clinical recommendations, assessed using the Guideline-Concordant Low Back Pain Vignette (GCLBP-V) [33], which reflects the application of these educational constructs to standardized clinical scenarios. The Red Flags Knowledge Test (RF-KT) served as a pre-specified manipulation-check outcome to evaluate content specificity between the intervention and attention-control sessions. In addition, credibility, expectancy, masking-integrity, contamination, and intervention fidelity were evaluated as process measures to assess intervention implementation and internal validity. All instruments were administered at baseline, immediately post-intervention, and six-month follow-up, unless otherwise specified. Demographic variables (year of study, age, and gender) were also recorded.

### The Revised Neurophysiology of Pain Questionnaire (RNPQ)

The RNPQ is a 12-item validated measure of pain neurophysiological knowledge [31]. Each item is a closed-ended statement answered as ‘true,’ ‘false,’ or ‘undecided.’ Scoring is binary: correct responses receive 1 point; incorrect or ‘undecided’ responses receive 0. Total scores range from 0 to 12, with higher scores indicating greater knowledge. The RNPQ has demonstrated excellent test-retest reliability (ICC = 0.97; 95% CI 0.93–0.99) and good internal consistency (Cronbach’s α = 0.84) [31]. The minimal clinically important difference (MCID) was predefined as an increase of ≥ 7.3% (equivalent to ≥ 1 point), based on established benchmarks for health professional student samples [22].

### The 13‑item modified Health Care Providers’ Pain and Impairment Relationship Scale (HC‑PAIRS)

The 13-item modified HC-PAIRS evaluates attitudes and beliefs toward individuals with chronic low back pain and their functional capacity [32]. Items are rated on a 7-point Likert scale (strongly disagree = 1; strongly agree = 7). Total scores range from 13 to 91, with lower scores indicating more positive, biopsychosocial-aligned attitudes. The scale has strong psychometric properties, including excellent internal consistency (Cronbach’s α = 0.92) and good test–retest reliability (ICC = 0.84; 95% CI, 0.78–0.89) [34]. The MCID was set a priori at a reduction of ≥ 4.6% (equivalent to 4 points), based on data validated for health professional students [22].

### Guideline-Concordant Low Back Pain Vignette (GCLBP-V)

The Guideline-Concordant Low Back Pain Vignette (GCLBP-V) is a vignette-based instrument developed to assess guideline-concordant clinical recommendations for non-specific low back pain across four management domains: daily activities, work participation, exercise, and bed rest. The instrument was independently developed based on contemporary international low back pain guidelines [5, 23–26] and uses a standardized case scenario with multiple-choice responses.

Responses are scored dichotomously (guideline-concordant = 1; non-concordant = 0), yielding a total score ranging from 0 to 4, with higher scores indicating greater alignment with evidence-based recommendations.

Prior to participant recruitment, the instrument underwent psychometric evaluation in an independent sample of undergraduate physiotherapy students, including assessment of content validity, construct validity, test–retest reliability, and measurement error, following methodology informed by the COSMIN (COnsensus-based Standards for the selection of health Measurement INstruments) framework [35]. The full development and psychometric validation study was subsequently published in a peer-reviewed journal [33].

### Red-Flags Knowledge Test (RF-KT)

The Red-Flags Knowledge Test (RF-KT) is a 10-item investigator-developed questionnaire designed to evaluate knowledge of red-flag screening for serious spinal pathology in low back pain (Additional file 1: Appendix S2). Items were derived from contemporary clinical guidelines and systematic reviews addressing red-flag recognition and referral decision-making [5, 23–29].

Items adopted a binary response format (“yes”/“no”), with correct responses scored as 1 and incorrect responses scored as 0. Total scores ranged from 0 to 10, with higher scores indicating greater knowledge of red-flag screening principles.

Formal psychometric validation was not undertaken because the RF-KT was developed primarily as an attention-control manipulation check to evaluate intervention specificity between the PNE and control educational sessions, rather than as a standalone outcome measure.

### Sample size calculation

An a priori sample size calculation was conducted using Stata/IC 17 (StataCorp LLC, College Station, TX). The calculation was based on the minimal clinically important difference (MCID) for the Health Care Providers’ Pain and Impairment Relationship Scale (HC-PAIRS), following the approach adopted in previous student-focused pain neuroscience education (PNE) trials [14, 22]. To estimate outcome variability, local preliminary psychometric data from undergraduate physiotherapy students were used [33]. Because the present trial enrolled only first- and second-year students, a standard deviation of 7 points was selected as a conservative planning estimate, exceeding the variability observed among junior students in the local validation sample. Assuming a 4-point MCID, a two-sided significance level of 0.05, 80% power, and equal allocation between groups, a minimum sample of 100 participants was required. Consistent with Mankelow et al. [22], the target sample size was increased by 20% to allow for anticipated attrition during the six-month follow-up, resulting in a planned sample of 120 participants (60 per group). This sample size was also considered sufficient to detect a 1-point MCID in RNPQ scores [22].

### Statistical analysis

Data were analyzed using Stata/IC 17.0 (StataCorp LLC, College Station, TX). Statistical significance was set at *p* < 0.05, and all analyses were two-tailed.

Descriptive statistics were presented as means and standard deviations (SDs) for continuous variables (age, RNPQ, HC-PAIRS, RF-KT) and as frequencies and percentages for categorical variables (gender, year of study, vignette responses).

Normality of continuous outcomes was examined using the Shapiro–Wilk test and graphical inspection. The primary continuous outcomes (RNPQ and HC-PAIRS) were analyzed using linear mixed-effects models with fixed effects for group (PNE vs. Red-flags), time (baseline, post-intervention, and six-month follow-up), and the Group × Time interaction. Models were adjusted for age, gender, and year of study. These covariates were selected a priori because they were considered plausible demographic and educational factors that could influence responses to the educational intervention. Collinearity among covariates was assessed before model fitting, and no problematic collinearity was identified. Participant-specific random intercepts were included to account for within-participant correlation across repeated measurements. Linear mixed-effects models were estimated using restricted maximum likelihood (REML), assuming compound symmetry. Observed group means and standard deviations (SDs) were presented descriptively at each time point. Prespecified between-group contrasts at post-intervention and six-month follow-up were derived from the mixed-effects models and expressed as adjusted mean differences (B) with 95% confidence intervals (CIs). Baseline values were presented descriptively without inferential comparisons because randomization occurred after baseline assessment.

The four GCLBP-V domains were analyzed separately, with guideline-concordant versus non-concordant responses treated as binary outcomes. Because the primary inferential interest concerned post-intervention and six-month guideline-concordant clinical recommendations, baseline domain responses were included as covariates rather than analyzed as repeated outcome measurements. Logistic mixed-effects models with a binomial logit link and participant-specific random intercepts were fitted for post-intervention and six-month responses, including fixed effects for group, time, and their interaction, and adjusting for baseline domain response, age, gender, and year of study. Observed proportions were presented descriptively at each time point, whereas post-intervention and six-month between-group comparisons were expressed as adjusted odds ratios (ORs) with 95% confidence intervals (CIs).

RF-KT scores were analyzed using linear mixed-effects models with fixed effects for group, time, and the Group × Time interaction, adjusted for age, gender, and year of study. Participant-specific random intercepts were included to account for repeated measurements across baseline, post-intervention, and six-month follow-up. Observed group means (SDs) were presented descriptively, whereas between-group comparisons at post-intervention and six-month follow-up were expressed as adjusted mean differences (B) with 95% confidence intervals (CIs).

To enhance interpretability, responder analyses were performed. A positive response was defined a *priori* using MCID thresholds informed by previous student-focused PNE research [22]. Separate multivariable logistic regression models were fitted for the post-intervention and six-month follow-up assessments, adjusting for age, gender, and year of study, to estimate adjusted odds ratios (ORs) with 95% CIs. Numbers needed to treat (NNTs) were calculated from model-predicted probabilities.

Credibility/expectancy ratings and masking/contamination indicators were compared between groups using t-tests or χ²/Fisher’s exact tests, as appropriate, and interpreted descriptively. The two primary outcomes (RNPQ and HC-PAIRS) were prespecified a *priori* and analyzed using separate linear mixed-effects models, each representing a distinct educational construct. Because the primary and secondary outcomes measured distinct educational constructs, no formal multiplicity adjustment was applied across outcome domains. Accordingly, findings from the secondary outcome analyses were interpreted as supportive rather than confirmatory. Because no missing data or attrition occurred, imputation was unnecessary.

## Results

**Fig. 2:**
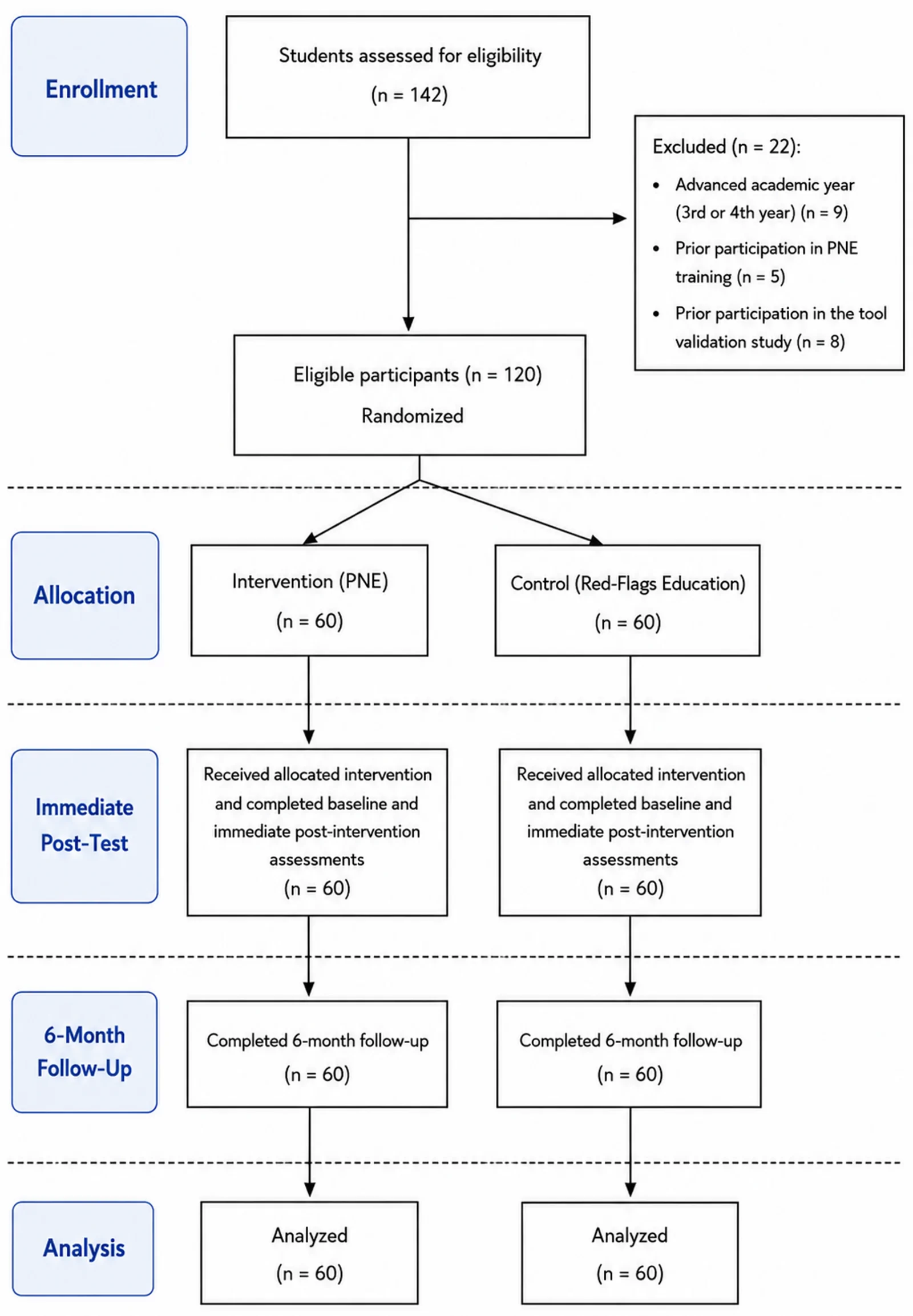
CONSORT flow diagram of participant enrollment, allocation, immediate post-intervention assessment, 6-month follow-up, and analysis. PNE, Pain Neuroscience Education

A total of 120 undergraduate physiotherapy students met the eligibility criteria and were randomized (60 per group). Participant flow through the trial is presented in Fig. 2. All participants completed baseline, immediate post-intervention, and six-month follow-up assessments, resulting in no attrition or missing data. All randomized participants received their allocated intervention and were included in the analyses.

### Baseline characteristics

Baseline demographic characteristics and outcome measures for both groups are presented descriptively in Table 1.

**Table 1:** Baseline characteristics of participants by group

	PNE (*n* = 60)	Control (*n* = 60)
Age, years	19.0 (0.6)	19.0 (0.6)
Gender, *n (%)*		
Female	40 (66.7)	39 (65.0)
Male	20 (33.3)	21 (35.0)
Year of study, *n (%)*		
Year 1	34 (56.7)	24 (40.0)
Year 2	26 (43.3)	36 (60.0)
RNPQ (0–12)	5.5 (2.0)	6.0 (2.1)
HC-PAIRS (13–91)	55.6 (9.4)	54.9 (8.7)
RF-KT (0–10)	5.3 (2.6)	5.4 (2.1)
Guideline-concordant clinical recommendations, *n (%)*		
Daily Activity	36 (60.0)	28 (46.7)
Work	35 (58.3)	39 (65.0)
Exercise	37 (61.7)	41 (68.3)
Bed Rest	23 (38.3)	17 (28.3)

Data are presented as mean (standard deviation, SD) for continuous variables and *n* (%) for categorical variables

*PNE* Pain Neuroscience Education, *RNPQ* Revised Neurophysiology of Pain Questionnaire, *HC-PAIRS* Health Care Providers’ Pain and Impairment Relationship Scale, *RF-KT* Red-Flags Knowledge Test

### Primary outcomes: adjusted group differences over time

**Fig. 3:**
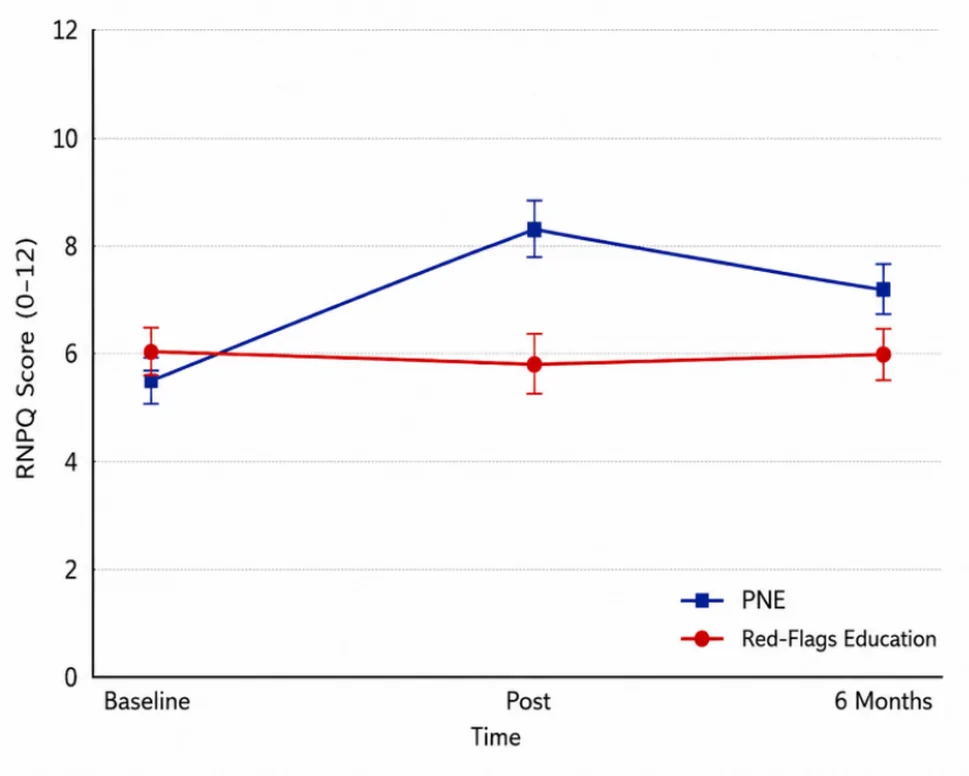
Mean Revised Neurophysiology of Pain Questionnaire (RNPQ) scores over time by group. Higher scores indicate greater pain neurophysiology knowledge. Error bars represent ± standard errors of the mean (SEM). PNE, Pain Neuroscience Education

**Fig. 4:**
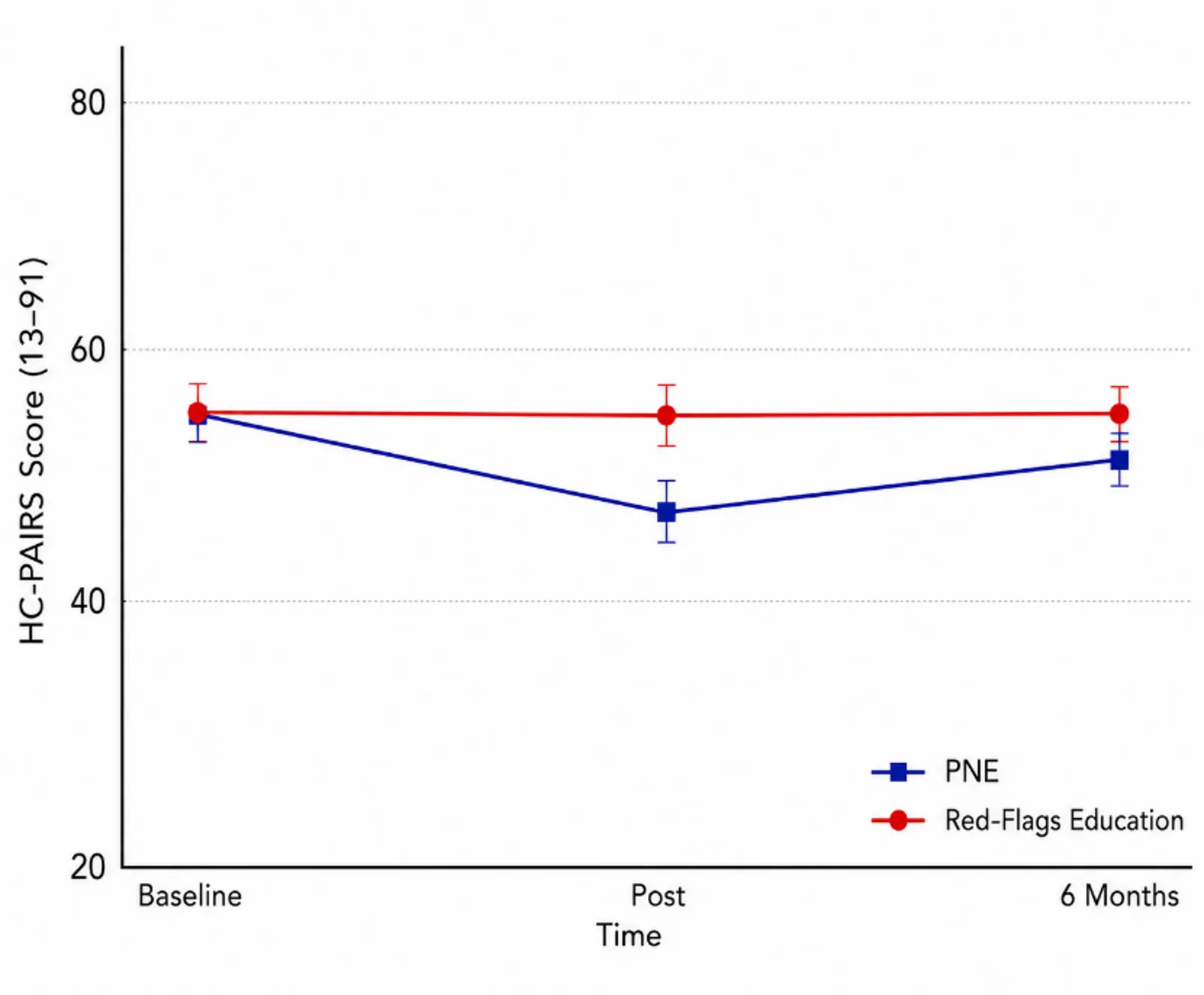
Mean Health Care Providers’ Pain and Impairment Relationship Scale (HC-PAIRS) scores over time by group. Lower scores indicate more favorable biopsychosocial pain-related attitudes. Error bars represent ± standard errors of the mean (SEM). PNE, Pain Neuroscience Education

Significant Group × Time interaction effects were observed for both RNPQ and HC-PAIRS (both *p* < 0.001) (Table 2). Immediately post-intervention, the PNE group demonstrated clinically meaningful and statistically significant improvements in pain neurophysiology knowledge and more favorable pain-related attitudes than the control group. At six-month follow-up, the adjusted between-group difference for RNPQ remained statistically significant (B = 1.20, 95% CI 0.25–2.15; *p* = 0.020), although the magnitude of the effect decreased from 2.46 to 1.20 points, indicating attenuation over time. The adjusted between-group difference for HC-PAIRS was no longer statistically significant (B = − 3.60, 95% CI − 7.25 to 0.05; *p* = 0.060), although scores remained numerically more favorable in the PNE group (Figs. 3 and 4). 

**Table 2:** Observed group means and adjusted between-group comparisons for RNPQ and HC-PAIRS over time

Outcome	PNE Mean (SD)	Control Mean (SD)	Adjusted Mean Difference (B) (95% CI)	*p*
RNPQ				
Baseline	5.5 (2.0)	6.0 (2.1)	—	—
Post	8.3 (1.9)	5.8 (2.0)	2.46 (1.68, 3.24)	**< 0.001**
6 months	7.2 (2.1)	6.0 (2.1)	1.20 (0.25, 2.15)	**0.020**
HC-PAIRS				
Baseline	55.6 (9.4)	54.9 (8.7)	—	—
Post	47.3 (7.5)	54.7 (8.6)	−7.54 (− 10.62, − 4.46)	**< 0.001**
6 months	51.9 (8.5)	54.8 (8.8)	−3.60 (− 7.25, 0.05)	0.060

Observed group means (SDs) are presented descriptively. Adjusted between-group mean differences (B) and 95% confidence intervals at post-intervention and six-month follow-up were derived from linear mixed-effects models including fixed effects for group, time, and the Group × Time interaction, adjusted for age, gender, and year of study, with participant-specific random intercepts. Baseline values are presented descriptively without inferential comparisons. For RNPQ, positive B values indicate higher pain knowledge in the PNE group relative to the control group, whereas for HC-PAIRS, negative B values indicate more favorable biopsychosocial attitudes in the PNE group relative to the control group

*PNE* Pain Neuroscience Education, *RNPQ* Revised Neurophysiology of Pain Questionnaire, *HC-PAIRS* Health Care Providers’ Pain and Impairment Relationship Scale, *CI* Confidence interval, *SD* Standard deviation

Bold *p*-values indicate *p* < 0.05

### Guideline-concordant clinical recommendations

Immediately post-intervention, students in the PNE group were significantly more likely to select guideline-concordant recommendations across all four clinical domains than students in the control group (adjusted ORs = 2.95–6.18; all *p* ≤ 0.015; Table 3), indicating clinically meaningful short-term improvements in guideline-concordant clinical recommendations. At six-month follow-up, these effects diminished and were no longer statistically significant (all *p* > 0.05), although the direction of the effects continued to favor the PNE group. This pattern is consistent with attenuation of the intervention effect on guideline-concordant clinical recommendations over time and mirrors the pattern observed for pain knowledge and pain-related attitudes.

**Table 3:** Guideline-concordant clinical recommendation responses by group and time

Domain	PNE % (*n*/*N*)	Control % (*n*/*N*)	Adjusted OR (95% CI)	*p*
Daily Activity				
Baseline	60.0 (36/60)	46.7 (28/60)	—	—
Post	75.0 (45/60)	53.3 (32/60)	2.95 (1.24, 7.02)	**0.015**
6 months	61.7 (37/60)	55.0 (33/60)	1.18 (0.53, 2.63)	0.688
Work				
Baseline	58.3 (35/60)	65.0 (39/60)	—	—
Post	85.0 (51/60)	55.0 (33/60)	6.16 (2.68, 14.18)	**< 0.001**
6 months	63.3 (38/60)	50.0 (30/60)	1.72 (0.80, 3.71)	0.165
Exercise				
Baseline	61.7 (37/60)	68.3 (41/60)	—	—
Post	86.7 (52/60)	53.3 (32/60)	6.18 (2.72, 14.03)	**< 0.001**
6 months	65.0 (39/60)	51.7 (31/60)	1.36 (0.65, 2.82)	0.416
Bed Rest				
Baseline	38.3 (23/60)	28.3 (17/60)	—	—
Post	81.7 (49/60)	58.3 (35/60)	4.58 (2.12, 9.93)	**< 0.001**
6 months	66.7 (40/60)	51.7 (31/60)	1.97 (0.94, 4.17)	0.074

Baseline values are presented descriptively without inferential comparisons. Percentages (%) represent students providing guideline-concordant responses within each domain. Adjusted odds ratios (ORs) and 95% confidence intervals for post-intervention and six-month follow-up were derived from logistic mixed-effects models including fixed effects for group, time, and the Group × Time interaction, adjusted for baseline domain response, age, gender, and year of study, with participant-specific random intercepts. ORs > 1 indicate greater odds of guideline-concordant recommendations in the PNE group relative to the control group.

*PNE* Pain Neuroscience Education, *OR* Odds ratio, *CI* Confidence interval

Bold *p*-values indicate *p* < 0.05

### Responder analysis

The PNE group demonstrated greater likelihood of clinically meaningful improvement, based on predefined minimal clinically important difference (MCID) thresholds, immediately post-intervention for both RNPQ (adjusted OR = 4.47, *p* < 0.001) and HC-PAIRS (adjusted OR = 3.23, *p* = 0.003). MCID thresholds were based on published evidence and have not been validated in Jordanian physiotherapy students. The corresponding numbers needed to treat (NNTs) of 2.7 and 3.6, respectively, indicated substantial educational benefit immediately post-intervention. At six-month follow-up, the intervention effects attenuated over time, with improvement remaining statistically significant for the RNPQ (adjusted OR = 2.48, *p* = 0.025) but not for the HC-PAIRS (adjusted OR = 1.69, *p* = 0.181) (Table 4).

**Table 4:** Responder analysis based on minimal clinically important difference (MCID) thresholds

Outcome	PNE Responders, *n* (%)	Control Responders, *n* (%)	Adjusted OR (95% CI)	*p*	NNT
RNPQ					
Post	41 (68.3)	19 (31.7)	4.47 (2.03, 9.82)	**< 0.001**	2.7
6 months	33 (55.0)	20 (33.3)	2.48 (1.12, 5.52)	**0.025**	4.5
HC-PAIRS					
Post	39 (65.0)	22 (36.7)	3.23 (1.48, 7.04)	**0.003**	3.6
6 months	30 (50.0)	23 (38.3)	1.69 (0.78, 3.65)	0.181	8.3

Responder proportions are presented as observed n (%) of participants achieving the predefined minimal clinically important difference (MCID). Adjusted odds ratios (ORs) and 95% confidence intervals were derived from multivariable logistic regression models adjusted for age, gender, and year of study. Numbers needed to treat (NNTs) were calculated from model-predicted probabilities. Lower NNT values indicate greater educational effectiveness of pain neuroscience education (PNE)

*PNE* Pain Neuroscience Education, *RNPQ* Revised Neurophysiology of Pain Questionnaire, *HC-PAIRS* Health Care Providers’ Pain and Impairment Relationship Scale, *OR* Odds ratio, *CI* Confidence interval, *NNT* Number needed to treat

Bold *p*-values indicate *p* < 0.05

### Manipulation check: intervention specificity

The Red-Flags Knowledge Test (RF-KT) was administered as a manipulation check to evaluate intervention specificity. The control group demonstrated significantly higher RF-KT scores than the PNE group immediately post-intervention (adjusted B = − 1.80, 95% CI − 2.53 to − 1.07; *p* < 0.001) and at six-month follow-up (adjusted B = − 1.45, 95% CI − 2.36 to − 0.54; *p* = 0.002). These findings support the specificity of the attention-matched control condition and minimal conceptual overlap between the educational interventions (Table 5).

**Table 5:** Manipulation check — Red-Flags Knowledge Test (RF-KT)

Time	PNE Mean (SD)	Control Mean (SD)	Adjusted B (95% CI)	*P*
Baseline	5.1 (2.2)	5.1 (2.1)	—	—
Post	5.3 (2.1)	7.1 (1.9)	−1.80 (− 2.53, − 1.07)	**< 0.001**
6 months	5.3 (2.2)	6.6 (2.2)	−1.45 (− 2.36, − 0.54)	**0.002**

Observed group means (SDs) are presented descriptively. Adjusted between-group mean differences (B) and 95% confidence intervals at post-intervention and six-month follow-up were derived from linear mixed-effects models including fixed effects for group, time, and the Group × Time interaction, adjusted for age, gender, and year of study, with participant-specific random intercepts. Baseline values are presented descriptively without inferential comparisons. Negative B values indicate lower RF-KT scores in the PNE group relative to the control group, reflecting greater knowledge acquisition in the attention-control (red-flags) group and supporting intervention specificity

*PNE* Pain Neuroscience Education, *RF-KT* Red-Flags Knowledge Test, *B* adjusted between-group mean difference, *CI* Confidence interval, *SD* Standard deviation.

Bold *p*-values indicate *p* < 0.05

### Credibility, expectancy, and masking integrity

Both the PNE and control groups reported similarly high credibility and expectancy ratings, with no between-group differences (Credibility: 7.3 (1.3) vs. 7.3 (1.4), *p* = 0.740; Expectancy: 7.1 (1.4) vs. 7.0 (1.5), *p* = 0.680) (Table 6). Only a small proportion of students in either group reported awareness of the study hypothesis (< 20%; *p* = 0.790), indicating low hypothesis awareness following the intervention. Fidelity monitoring demonstrated high protocol adherence (≥ 90% planned content delivered), and cross-group contamination remained low at both post-session and six-month follow-up assessments (≤ 10%, *p* > 0.05), supporting strong procedural fidelity. Detailed credibility, expectancy, masking-integrity, and contamination results are presented in Additional file 1: Table S2. 

**Table 6:** Intervention credibility, expectancy, and masking integrity

Measure	PNE	Control	*P*
Adapted CEQ Credibility (0–10)	7.3 (1.3)	7.3 (1.4)	0.740
Adapted CEQ Expectancy (0–10)	7.1 (1.4)	7.0 (1.5)	0.680
Hypothesis Awareness, n (%)	9 (15.0)	8 (13.3)	0.790

Credibility, expectancy, and masking-integrity measures were assessed immediately post-intervention. Continuous variables are presented as mean (SD), and categorical variables as *n* (%)

*CEQ* Credibility and Expectancy Questionnaire, *PNE* Pain Neuroscience Education

## Discussion

To our knowledge, this is the first randomized controlled trial (RCT) conducted in Jordan and one of the first from the Arab Middle East to evaluate pain neuroscience education (PNE) among undergraduate physiotherapy students. A single 70-minute PNE lecture substantially improved pain neurophysiology knowledge and shifted attitudes toward a more biopsychosocial framework among undergraduate physiotherapy students in Jordan. Compared with an attention-matched control, the PNE group demonstrated significantly higher RNPQ scores and lower HC-PAIRS scores immediately post-intervention. Although these educational gains attenuated over six months, pain knowledge improvements remained statistically and clinically meaningful, whereas the attitudinal shift was not sustained. Immediate benefits also extended to guideline-concordant clinical recommendations, as demonstrated by marked improvement in vignette-based responses, which similarly decayed by follow-up.

Immediately post-intervention, the adjusted between-group difference in RNPQ was 2.46 points, corresponding to a within-group improvement of approximately 23% (2.7 points) in the PNE group. This magnitude is broadly comparable to the within-group improvements of 20–22% reported in other single-session PNE studies among student cohorts [14, 15], although direct quantitative comparisons should be interpreted cautiously because previous studies have generally reported descriptive within-group changes rather than adjusted between-group estimates. The smaller 15% gains reported by Mankelow et al. [22] may reflect higher baseline pain knowledge in their mixed-discipline UK sample. Much larger improvements (> 40%) have been observed following more intensive multi-hour PNE workshops [36, 37]; however, these interventions are not directly comparable due to differences in intervention intensity and educational design.

Immediately post-intervention, the adjusted between-group difference in HC-PAIRS was − 7.54 points, corresponding to a within-group reduction of 8.3 points in the PNE group. As with the RNPQ findings, comparisons with previous studies should be interpreted cautiously because published educational trials have generally reported descriptive within-group changes rather than adjusted between-group estimates. The observed within-group reduction was broadly consistent with the range reported following single-session PNE interventions, where immediate reductions typically range from 10 to 18 points. Colleary et al. [14] observed a reduction of 15 points, while Mankelow et al. [22] and Maguire et al. [15] reported nearly twice the reduction (17.1 and 18 points, respectively) seen in our cohort, despite comparable baseline scores. A possible explanation for these larger effects lies in methodological factors in those earlier trials, including smaller and more heterogeneous samples, which can yield less stable and larger effect estimates, and a lack of formal delivery checks to ensure intervention fidelity or control for contamination. In contrast, multi-hour curricula or program-wide pain-education exposures have demonstrated more modest reductions of approximately 9–10 points [38, 39], and some abbreviated formats have produced no attitudinal shift [36]. Despite this variability, the observed improvement exceeded the a priori MCID threshold and supports recent syntheses concluding that short, well-targeted PNE is capable of eliciting meaningful, immediate attitudinal change in student cohorts [12].

An additional consideration is that the educational emphasis differed between the two interventions. Whereas the attention-control lecture primarily reinforced factual knowledge related to red-flag screening for serious spinal pathology, the PNE intervention emphasized conceptual reframing through a contemporary biopsychosocial understanding of pain. Consequently, some of the observed differences in pain-related attitudes may reflect the educational impact of conceptual reframing itself, in addition to the specific content of PNE. Nevertheless, the use of an attention-matched, clinically relevant comparator strengthened internal validity by controlling for non-specific effects associated with educator contact, lecture duration, participant engagement, and attention. Future studies comparing PNE with other conceptually oriented educational interventions may help further clarify the specificity of these educational effects.

The six-month follow-up, the magnitude of the adjusted between-group effects was smaller than immediately post-intervention across the study outcomes. The adjusted between-group differences for both RNPQ and HC-PAIRS attenuated over time, with the RNPQ advantage decreasing from 2.46 to 1.20 points and the HC-PAIRS advantage from 7.54 to 3.60 points. Consequently, the between-group difference remained statistically significant for RNPQ but was no longer statistically significant for HC-PAIRS. Similarly, guideline-concordant recommendations showed a consistent downward trend, with no domain maintaining statistical significance. This attenuation pattern mirrors findings by Mankelow et al. [22], where immediate gains in knowledge and attitudes decreased substantially by six months, with no sustained between-group differences observed in vignette-based recommendations, likely influenced by ceiling effects. A similar consideration may apply to the present study, particularly for the GCLBP-V domains, where immediate post-intervention guideline-concordant responses exceeded 80% in several domains, potentially limiting sensitivity to detect sustained between-group differences at six months. In contrast, ceiling effects are less likely to have substantially influenced the RNPQ findings because post-intervention scores remained well below the maximum possible score. Nevertheless, studies evaluating broader curriculum-level or repeated pain education have demonstrated greater durability, with knowledge gains maintained at six months [16]. In addition, more positive attitudes toward pain and function have been observed among senior physiotherapy students following extended undergraduate training exposure [38, 39]. Collectively, these findings suggest that while single-session PNE consistently produces large short-term educational effects, lasting improvements in knowledge, beliefs, and guideline-concordant recommendations may require recursive reinforcement of pain science content throughout undergraduate curricula [22, 37].

This study also employed the MCID-based responder analysis to enhance practical interpretability of the findings. Immediately post-intervention, the Number Needed to Treat (NNT) figures indicated high educational efficiency. The NNT values of 2.7 for pain knowledge and 3.6 for attitudes suggest that few students needed to receive the PNE intervention for one to achieve a clinically meaningful benefit. Although these effects diminished at six months (with NNTs increasing to 4.5 for knowledge and 8.3 for attitudes), a pattern consistent with the observed attenuation, the knowledge benefit remained statistically significant at six months. The absence of NNT data for the RNPQ and HC-PAIRS in the PNE literature, a gap identified by [12], highlights the novel contribution of our analysis in interpreting the educational efficiency of brief PNE interventions.

The concurrent improvements observed across knowledge, beliefs, and vignette-based clinical recommendations are consistent with the cognitive and behavioral mechanisms through which PNE is theorized to exert its educational effects. Conceptually, PNE is proposed to replace maladaptive biomedical interpretations with a modern, neurophysiologically grounded biopsychosocial understanding of pain [13, 38]. This process is theorized to involve correcting misconceptions and restructuring learners’ mental models of pain, shifting them toward viewing pain as a protective output of the nervous system modulated by interactive psychological and contextual factors [39]. This conceptual shift has been associated with favorable changes in attitudes and beliefs, including reduced fear-avoidance and increased acceptance of activity despite pain [40], which may contribute to more guideline-concordant clinical recommendations [12]. Our findings of improvement in knowledge, attitudes, and guideline-concordant recommendations are broadly consistent with the cognitive-affective-behavioral mechanisms proposed within contemporary pain neuroscience education frameworks [39, 40]. Nevertheless, because guideline-concordant recommendations were assessed using a standardized vignette rather than observed clinical behavior, the relationship between these educational gains and real-world clinical decision-making remains to be established. Collectively, these findings are consistent with the proposition that even a single PNE session may initiate meaningful educational changes during early physiotherapy training.

These findings confirm targeted PNE’s role as an effective foundation for undergraduate pain education. In contrast, the observed attenuation of gains highlights the limitations of one-off PNE sessions and underscores the necessity of achieving sustained competency through the recursive reinforcement of pain science within a scaffolded curriculum. Strategies such as clinical application, reflective practice, and interdisciplinary teaching may support long-term retention and clinical transfer [16, 41, 42]. Embedding PNE longitudinally throughout core curricula aligns with the International Association for the Study of Pain (IASP) frameworks for pre-licensure health-professional education [43] and is particularly germane in contexts like Jordan. This integrated approach may better prepare graduates to apply evidence-informed biopsychosocial principles when managing musculoskeletal pain.

Although both educational sessions were delivered by the same educator, which may limit the ability to completely distinguish intervention-specific effects from educator-specific influences, several design features were incorporated to minimize this potential source of bias. The same educator delivered both interventions using standardized teaching materials, equivalent session duration, and predefined instructional protocols, while independent fidelity monitoring confirmed high protocol adherence. Furthermore, comparable credibility and expectancy ratings between groups suggest that differential participant engagement or perceived educational value was unlikely to fully account for the observed between-group differences. Nevertheless, future multi-institutional trials involving multiple educators are warranted to determine whether these findings are reproducible across different instructors and teaching contexts.

This study offers several methodological and contextual strengths to the pain education literature. To our knowledge, this is the first randomized controlled trial conducted among undergraduate physiotherapy students in Jordan and one of the first from the Arab Middle East evaluating pain neuroscience education. Moreover, the study’s MCID-based responder analysis quantified clinically meaningful change, offering a clearer measure of educational impact than group means alone. The inclusion of an attention-matched control, alongside masking-integrity, credibility/expectancy, and manipulation checks, further enhanced internal validity and strengthened the study’s rigor for informing pain education research and curricula. The complete six-month follow-up was likely facilitated by embedding outcome assessments within scheduled compulsory teaching sessions and supplementing these with routine reminders. Although this strategy may not be feasible in all educational settings, it represents a pragmatic approach that may help maximize participant retention in similar educational trials.

Despite its strengths, several limitations should be acknowledged. First, while the inclusion of a six-month follow-up allowed evaluation of short-term retention and attenuation patterns, the absence of an intermediate follow-up assessment (e.g., three months) limited the ability to determine when attenuation in pain knowledge, attitudes, and guideline-concordant clinical recommendations first emerged. In addition, the six-month follow-up cannot confirm the durability of educational effects beyond that period. Second, because interventions were delivered in a single group session, session-level factors potentially confounded intervention effects. This was mitigated by using an attention-matched control, standardized materials, fidelity monitoring, and checks confirming equivalent group credibility and low awareness of the study hypothesis. Third, despite being a psychometrically validated instrument, the GCLBP-V remains a vignette-based measure of clinical recommendations rather than observed clinical behavior or patient outcomes. Therefore, the findings should be interpreted as educational outcomes rather than direct evidence of changes in clinical practice. In addition, although the primary and secondary outcomes were specified a priori, no formal multiplicity adjustment was applied across the co-primary outcomes, prespecified follow-up contrasts, or secondary outcome domains. This may have increased the risk of type I error. Accordingly, statistically significant findings from the secondary outcome analyses should be interpreted cautiously and considered supportive rather than confirmatory. Fourth, although use of the same educator enhanced intervention standardization, educator-specific influences cannot be entirely excluded. Standardized teaching materials, fidelity monitoring, and comparable credibility and expectancy ratings likely minimized this risk; nevertheless, replication using multiple educators is warranted. Fifth, because participation was voluntary, self-selection bias cannot be excluded. Sixth, the single-institution context limits the generalizability of the findings to other educational systems, health disciplines, or cultural contexts. Seventh, the MCID thresholds for RNPQ and HC-PAIRS were drawn from the best available literature but have not been validated specifically for Jordanian first- and second-year students; therefore, the NNT estimates should be interpreted with caution. Eighth, restricting recruitment to first- and second-year students reduced potential confounding from prior pain education and clinical training; however, the findings may not be generalizable to senior undergraduate physiotherapy students, whose greater clinical exposure and curricular experience may influence their responses to pain neuroscience education. Ninth, the Red-Flags Knowledge Test (RF-KT) was designed solely as an attention-control measure and was not psychometrically validated, which limits its interpretability beyond confirming intervention specificity. Tenth, expectancy was assessed only after the educational sessions. Although randomization and masking procedures were designed to minimize systematic differences in participant expectations before the intervention, baseline expectancy was not directly measured and therefore cannot be formally compared between groups. Finally, as the intervention was limited to a single session, longer-term or repeated exposures may produce different retention patterns.

## Conclusions

This randomized controlled trial demonstrated that a single 70-minute pain neuroscience education (PNE) lecture produced significant immediate improvements in Jordanian physiotherapy students’ pain knowledge, pain-related attitudes, and guideline-concordant clinical recommendations. Although some intervention effects attenuated over six months, improvements in pain knowledge remained statistically and educationally meaningful. These findings support consideration of embedding PNE early within pre-licensure physiotherapy curricula, particularly during the early stages of undergraduate training, while future studies should determine whether similar effects are observed in more senior students and across different institutions. The methodological rigor of this trial, including the use of a validated vignette-based outcome measure, MCID-based responder analyses, and robust masking-integrity and fidelity procedures, enhances confidence in the internal validity and reliability of the findings. Future research should investigate multi-session and longitudinal PNE approaches, evaluate their integration within broader physiotherapy curricula, and determine whether these findings are reproducible across different institutions, learner populations, and health-professional disciplines, and whether educational gains translate into sustained clinical behaviors and improved patient outcomes. Such studies are needed to establish the generalizability of these findings.

## Supplementary Information

Additional file 1: Supplementary materials. Table S1: Adapted CEQ, masking-integrity, and contamination items. Table S2: Credibility, expectancy, masking-integrity, and contamination outcomes. Appendix S1: Intervention fidelity checklist. Appendix S2: Red Flags Knowledge Test (RF-KT).

## Data Availability

The datasets generated and/or analyzed during the current study are available from the corresponding author on reasonable request.

## Abbreviations

CEQ: Credibility and Expectancy Questionnaire
CI: Confidence Interval
COSMIN: COnsensus-based Standards for the selection of health Measurement INstruments
GCLBP-V: Guideline-Concordant Low Back Pain Vignette
HC-PAIRS: Health Care Providers’ Pain and Impairment Relationship Scale
IASP: International Association for the Study of Pain
ICC: Intraclass Correlation Coefficient
IRB: Institutional Review Board
LBP: Low Back Pain
MCID: Minimal Clinically Important Difference
NNT: Number Needed to Treat
OR: Odds Ratio
PNE: Pain Neuroscience Education
RA: Research Assistant
RCT: Randomized Controlled Trial
RF-KT: Red Flags Knowledge Test
RNPQ: Revised Neurophysiology of Pain Questionnaire
SD: Standard Deviation
SEM: Standard Error of the Mean

## References

[1] Briggs AM, Woolf AD, Dreinhöfer K, et al. Reducing the global burden of musculoskeletal conditions. Bull World Health Organ. 2018;96(5):366–8. 10.2471/BLT.17.204891. PMC5985424 29875522

[2] Rice ASC, Smith BH, Blyth FM. Pain and the global burden of disease. Pain. 2016;157(4):791–6. 10.1097/j.pain.0000000000000454. 26670465

[3] Mose S, Kent P, Smith A, Andersen JH, Christiansen DH. Trajectories of Musculoskeletal Healthcare Utilization of People with Chronic Musculoskeletal Pain - A Population-Based Cohort Study. Clin Epidemiol. 2021;13:825–43. 10.2147/CLEP.S323903. PMC8455515 34557040

[4] Mills SEE, Nicolson KP, Smith BH. Chronic pain: a review of its epidemiology and associated factors in population-based studies. Br J Anaesth. 2019;123(2):e273–83. 10.1016/j.bja.2019.03.023. PMC6676152 31079836

[5] National Institute for Health and Care Excellence. Low back pain and sciatica in over 16s: assessment and management (NG59). London: National Institute for Health and Care Excellence. 2016. Updated 2020. Available from: https://www.nice.org.uk/guidance/ng59. Accessed 22 Feb 2025.

[6] Darlow B, Fullen BM, Dean S, Hurley DA, Baxter GD, Dowell A. The association between health care professional attitudes and beliefs and the attitudes and beliefs, clinical management, and outcomes of patients with low back pain: a systematic review. Eur J Pain. 2012;16(1):3–17. 10.1016/j.ejpain.2011.06.006. 21719329

[7] Leysen M, Nijs J, Van Wilgen P, et al. Attitudes and beliefs on low back pain in physical therapy education: a cross-sectional study. Braz J Phys Ther. 2021;25(3):319–28. 10.1016/j.bjpt.2020.08.002. PMC8134791 32847758

[8] Houben RM, Ostelo RW, Vlaeyen JW, Wolters PM, Peters M, Stomp-van den Berg SG. Health care providers’ orientations towards common low back pain predict perceived harmfulness of physical activities and recommendations regarding return to normal activity. Eur J Pain. 2005;9(2):173–83. 10.1016/j.ejpain.2004.05.002. 15737810

[9] Bishop A, Foster NE. Do physical therapists in the United Kingdom recognize psychosocial factors in patients with acute low back pain? Spine (Phila Pa 1976). 2005;30(11):1316–22. 10.1097/01.brs.0000163883.65321.33. 15928559

[10] Briggs EV, Carr ECJ, Whittaker MS. Survey of undergraduate pain curricula for healthcare professionals in the United Kingdom. Eur J Pain. 2011;15(8):789–95. 10.1016/j.ejpain.2011.01.006. 21330174

[11] Briggs EV, Battelli D, Gordon D, et al. Current pain education within undergraduate medical studies across Europe: advancing the provision of pain education and learning (APPEAL) study. BMJ Open. 2015;5(8):e006984. 10.1136/bmjopen-2014-006984. PMC4538268 26260345

[12] Mankelow J, Ryan C, Taylor P, Atkinson G, Martin D. A systematic review and meta-analysis of the effects of biopsychosocial pain education upon health care professional pain attitudes, knowledge, behavior and patient outcomes. J Pain. 2022;23(1):1–24. 10.1016/j.jpain.2021.06.010. 34237464

[13] Butler DS, Moseley GL. Explain pain. 2nd ed. Adelaide, Australia: Noigroup; 2013.

[14] Colleary G, O’Sullivan K, Griffin D, Ryan CG, Martin DJ. Effect of pain neurophysiology education on physiotherapy students’ understanding of chronic pain, clinical recommendations and attitudes towards people with chronic pain: a randomised controlled trial. Physiotherapy. 2017;103(4):423–9. 10.1016/j.physio.2017.01.006. 28797666

[15] Maguire N, Chesterton P, Ryan C. The effect of pain neuroscience education on sports therapy and rehabilitation students knowledge, attitudes, and clinical recommendations toward athletes with chronic pain. J Sport Rehabil. 2019;28(5):438–43. 10.1123/jsr.2017-0212. 29405811

[16] Saracoglu I, Aksoy CC, Afsar E, Arik MI. Does pain neuroscience education improve pain knowledge, beliefs and attitudes in undergraduate physiotherapy students? Physiother Res Int. 2021;26(2):e1898. 10.1002/pri.1898. 33528084

[17] Aldera M. Pain neuroscience education training enhances pain knowledge among a sample of Saudi physiotherapists but it does not alter attitudes or beliefs. Int J Phys Therapy Res Pract. 2024;3:396–406. 10.62464/ijoprp.v3i10.57.

[18] Muhsen A, Madi M, Wshah A, et al. Pain knowledge, attitudes, and beliefs among final-year physiotherapy and occupational therapy students in Jordan: a cross-sectional study. J Pain Res. 2025;18:6243–57. 10.2147/JPR.S565885. PMC12649783 41311853

[19] Boutron I, Estellat C, Guittet L, et al. Methods of blinding in reports of randomized controlled trials assessing pharmacologic treatments: a systematic review. PLoS Med. 2006;3(10):e425. 10.1371/journal.pmed.0030425. PMC1626553 17076559

[20] Boutron I, Moher D, Altman DG, Schulz KF, Ravaud P, CONSORT Group. Extending the CONSORT statement to randomized trials of nonpharmacologic treatment: explanation and elaboration. Ann Intern Med. 2008;148(4):295–309. 10.7326/0003-4819-148-4-200802190-00008. 18283207

[21] Devilly GJ, Borkovec TD. Psychometric properties of the credibility/expectancy questionnaire. J Behav Ther Exp Psychiatry. 2000;31(2):73–86. 10.1016/s0005-7916(00)00012-4. 11132119

[22] Mankelow J, Ryan C, Taylor P, Martin D. The effect of pain neurophysiology education on healthcare students’ knowledge, attitudes and behaviours towards pain: a mixed-methods randomized controlled trial. Musculoskelet Sci Pract. 2020;50:102249. 10.1016/j.msksp.2020.102249. 32920228

[23] Qaseem A, Wilt TJ, McLean RM, et al. Noninvasive Treatments for Acute, Subacute, and Chronic Low Back Pain: A Clinical Practice Guideline From the American College of Physicians. Ann Intern Med. 2017;166(7):514–30. 10.7326/M16-2367. 28192789

[24] George SZ, Fritz JM, Silfies SP, et al. Interventions for the management of acute and chronic low back pain: revision 2021. J Orthop Sports Phys Ther. 2021;51:CPG1–60. 10.2519/jospt.2021.0304. PMC10508241 34719942

[25] U.S. Department of Veterans Affairs, U.S. Department of Defense. VA/DoD Clinical Practice Guideline for the Diagnosis and Treatment of Low Back Pain. Version 3.0. Washington, DC: U.S. Department of Veterans Affairs and U.S. Department of Defense. 2022. Available from: https://www.healthquality.va.gov/guidelines/Pain/lbp/. Accessed 22 Feb 2025.

[26] Patel ND, Broderick DF, Burns J, et al. ACR appropriateness criteria low back pain. J Am Coll Radiol. 2016;13:1069–78. 10.1016/j.jacr.2016.06.008. 27496288

[27] Finucane LM, Downie A, Mercer C, et al. International Framework for Red Flags for Potential Serious Spinal Pathologies. J Orthop Sports Phys Ther. 2020;50(7):350–72. 10.2519/jospt.2020.9971. 32438853

[28] Downie A, Williams CM, Henschke N, et al. Red flags to screen for malignancy and fracture in patients with low back pain: systematic review. BMJ. 2013;347:f7095. 10.1136/bmj.f7095. PMC3898572 24335669

[29] Williams CM, Henschke N, Maher CG, et al. Red flags to screen for vertebral fracture in patients presenting with low-back pain. Cochrane Database Syst Rev. 2023;11:CD008643. 10.1002/14651858.CD008643.pub3. PMC10683370 38014846

[30] Bellg AJ, Borrelli B, Resnick B, et al. Enhancing treatment fidelity in health behavior change studies: best practices and recommendations from the NIH Behavior Change Consortium. Health Psychol. 2004;23(5):443–51. 10.1037/0278-6133.23.5.443. 15367063

[31] Catley MJ, O’Connell NE, Moseley GL. How good is the neurophysiology of pain questionnaire? A Rasch analysis of psychometric properties. J Pain. 2013;14:818–27. 10.1016/j.jpain.2013.02.008. 23651882

[32] Houben RM, Vlaeyen JW, Peters M, et al. Health care providers’ attitudes and beliefs towards common low back pain: factor structure and psychometric properties of the HC-PAIRS. Clin J Pain. 2004;20:37–44. 10.1097/00002508-200401000-00008. 14668655

[33] Muhsen A, Abudoush AN, Bin Sheeha B, Darabseh MZ, Shallan A, Wshah A, Eilayyan OJ. Development and psychometric validation of the Guideline-Concordant Low Back Pain Vignette (GCLBP-V) in undergraduate physiotherapy students. J Pain Res. 2026;19:623929. 10.2147/JPR.S623929. PMC13355654 42436827

[34] Moran RW, Rushworth WM, Mason J. Investigation of four self-report instruments (FABT, TSK-HC, Back-PAQ. HC-PAIRS) to measure healthcare practitioners’ attitudes and beliefs toward low back pain: reliability, convergent validity and survey of New Zealand osteopaths and manipulative physiotherapists. Musculoskelet Sci Pract. 2017;32:44–50. 10.1016/j.msksp.2017.08.008. 28858681

[35] Terwee CB, Prinsen CAC, Chiarotto A, et al. COSMIN methodology for evaluating the content validity of patient-reported outcome measures: a Delphi study. Qual Life Res. 2018;27(5):1159–70. 10.1007/s11136-018-1829-0. PMC5891557 29550964

[36] Cox T, Louw A, Puentedura EJ. An abbreviated therapeutic neuroscience education session improves pain knowledge in first-year physical therapy students but does not change attitudes or beliefs. J Man Manip Ther. 2017;25:11–21. 10.1080/10669817.2015.1122308. PMC5539573 28855788

[37] Smart KM. Pain science and practice as a threshold concept within undergraduate and pre-registration physiotherapy education: a jewel of the curriculum? BMC Med Educ. 2023;23:732. 10.1186/s12909-023-04733-z. PMC10559438 37803373

[38] Moseley L. Unraveling the barriers to reconceptualization of the problem in chronic pain: the actual and perceived ability of patients and health professionals to understand the neurophysiology. J Pain. 2003;4:184–9. 10.1016/S1526-5900(03)00488-7. 14622702

[39] Moseley GL, Leake HB, Beetsma AJ, et al. Teaching patients about pain: the emergence of pain science education, its learning frameworks and delivery strategies. J Pain. 2024;25:104425. 10.1016/j.jpain.2023.11.008. 37984510

[40] Adenis N, Gosselin K, Stetsenko N, Thevenon A. Clarification of the pain neuroscience education concept in the management of patients with persistent low back pain: a scoping review. J Back Musculoskelet Rehabil. 2023;36:995–1010. 10.3233/BMR-220370. 37458022

[41] Latimer J, Maher C, Refshauge K. The attitudes and beliefs of physiotherapy students to chronic back pain. Clin J Pain. 2004;20:45–50. 10.1097/00002508-200401000-00009. 14668656

[42] Ryan C, Murphy D, Clark M, Lee A. The effect of a physiotherapy education compared with a non-healthcare education on the attitudes and beliefs of students towards functioning in individuals with back pain: an observational, cross-sectional study. Physiotherapy. 2010;96:144–50. 10.1016/j.physio.2009.09.010. 20420961

[43] International Association for the Study of Pain. IASP Curriculum Outline on Pain for Physical Therapy. Washington, DC: International Association for the Study of Pain. 2018. Available from: https://www.iasp-pain.org. Accessed 22 Feb 2025.

