# Supplementary material for: The impact of pain neuroscience education on physical therapy students’ pain knowledge, attitudes, beliefs, and clinical recommendations: a randomized controlled trial
Source: BMC Med Educ. 2026 Jul 27;26:1698. doi: 10.1186/s12909-026-09994-y (PMC13689214; doi:10.1186/s12909-026-09994-y)
Supplement: Supplementary file 1 [file 12909_2026_9994_MOESM1_ESM.docx]

**Additional file 1. Supplementary Materials**

**The Impact of Pain Neuroscience Education on Physical Therapy Students’ Pain Knowledge, Attitudes, Beliefs, and Clinical Recommendations: A Randomized Controlled Trial**

**Contents	Page**

**Table S1. Adapted CEQ, Masking-Integrity, and Contamination Items	3-4**

**Table S2. Credibility, Expectancy, Masking-Integrity, and Contamination	Outcomes	5**

**Appendix S1. Intervention Fidelity Checklist	6-8**

**Appendix S2. Red Flags Knowledge Test (RF-KT)	9**

**Table S1.**

**Adapted CEQ, Masking-Integrity, and Contamination Items**

Items Q1–Q6 were adapted from the Credibility/Expectancy Questionnaire (CEQ) originally developed by Devilly and Borkovec [1]. Wording was modified to reflect an educational context (e.g. “therapy” replaced with “lecture”), while preserving the original credibility and expectancy constructs. Items Q7–Q13 were study-specific and designed to assess allocation guess, confidence, hypothesis awareness, and cross-group contamination.

| Section | Item / Question |
| --- | --- |
| **A. Credibility** (CEQ – adapted) | 1. How logical did the lecture content appear to you?  *0 = Not at all logical; 10 = Extremely logical* |
|  | 2. How confident are you that what you learned will improve your understanding of pain?  *0 = Not at all confident; 10 = Extremely confident* |
|  | 3. How believable did you find the explanations provided during the lecture?  *0 = Not at all believable; 10 = Completely believable* |
| **B. Expectancy** (CEQ – adapted) | 4. How much do you expect this lecture will change how you think about pain in future practice?  0 *= Not at all; 10 = A great deal* |
|  | 5. How much improvement do you expect in your pain-related knowledge because of this lecture?  *0 = No improvement; 10 = A great deal of improvement* |
|  | 6. Overall, how much benefit do you expect from this lecture?  *0 = No benefit; 10 = Exceptional benefit* |
| **C. Allocation Guess / Masking** | 7. Which topic do you think your lecture *emphasized most*?  ☐ Pain neurophysiology ☐ Both equally ☐ Red flags for musculoskeletal pain ☐ Not sure |
|  | 8. How confident are you in your answer above?  0 = Not confident; 10 = Completely confident |
|  | 9. Before today, did you know there were two different lectures being delivered in this study?  ☐ Yes ☐ No ☐ Not sure |
|  | 10. Did you have any idea what the study hypothesis was (e.g., which lecture was expected to be better)?  ☐ Yes ☐ No ☐ Not sure |
| **D. Contamination/ Information Sharing** | 11. Have you discussed the lecture content with students from the other group?  ☐ Yes ☐ No |
|  | 12. If yes, please describe briefly (optional).___________________ |
|  | 13. At the 6-month follow-up: have you attended or read additional materials related to pain neurophysiology or red flags?  ☐ Yes ☐ No |

**Notes:**
The adapted CEQ was used descriptively as a credibility, expectancy, and masking-integrity measure rather than as a psychometric outcome measure. Study-specific items assessed masking integrity and contamination. No validated cut-points were assumed; interpretation focused on between-group equivalence and low levels of hypothesis awareness and contamination.

**References**

1. Devilly GJ, Borkovec TD. Psychometric properties of the credibility/expectancy questionnaire. J Behav Ther Exp Psychiatry. 2000;31(2):73-86. doi:10.1016/s0005-7916(00)00012-4

Table S2. Credibility, expectancy, masking integrity, and contamination measures

| **Variable** | **Time** | **PNE Mean (SD) or n %** | **Control Mean (SD) or n %** | **Mean / Proportion Difference (95% CI)** | **p** |
| --- | --- | --- | --- | --- | --- |
| Adapted CEQ Credibility (0–10) | Post | 7.3 (1.3) | 7.3 (1.4) | 0.06 (−0.45, 0.57) | 0.740 |
| Adapted CEQ Expectancy (0–10) | Post | 7.1 (1.4) | 7.0 (1.5) | 0.13 (−0.48, 0.74) | 0.680 |
| Allocation guess confidence (0–10) | Post | 5.1 (2.0) | 4.9 (2.0) | 0.26 (−0.53, 1.05) | 0.514 |
| Allocation response:  “Not sure” | Post | 33 (55.0) | 32 (53.3) | 1.7% (−17.2, 20.6) | 0.840 |
| Aware of two study arms (Yes) | Post | 12 (20.0) | 10 (16.7) | 3.3% (−10.5, 17.2) | 0.622 |
| Hypothesis awareness (Yes) | Post | 9 (15.0) | 8 (13.3) | 1.7% (−11.3, 14.7) | 0.790 |
| Reported Contamination | Post | 3 (5.0) | 4 (6.7) | −1.7% (−12.0, 8.7) | 0.705 |
|  | 6 months | 5 (8.3) | 6 (10.0) | −1.7% (−13.6, 10.2) | 0.765 |

Continuous variables are presented as mean (SD), and categorical variables as n (%). Mean differences are reported for continuous variables and proportion differences for categorical variables, each with 95% confidence intervals. Group comparisons used independent *t*-tests for continuous variables and χ² or Fisher’s exact tests for categorical variables, as appropriate. Contamination was reassessed at six-month follow-up. CEQ, Credibility and Expectancy Questionnaire; CI, confidence interval; PNE, Pain Neuroscience Education; SD, standard deviation.

**Appendix S1.**

**Predefined Intervention Fidelity Checklist**

**Session start time: ________ End time: ________**

**SECTION 1 – Planned Content Delivery (Primary Fidelity Criterion)**

**Instruction:**
Tick **Yes/No** based on whether each planned content item was *substantively covered* as per the script. Use comments for partial or modified delivery.

**A. If this is a PNE session (leave blank for Red-flags sessions)**

| **PNE Item** | **Planned PNE Content Component** | **Delivered as Planned? (No = 0, Yes = 1)** |
| --- | --- | --- |
| 1. | Overview of modern pain concepts and contemporary pain neuroscience education framework | ☐ 0 ☐ 1 |
| 2. | Basic nociceptive physiology (nociceptors, transmission, modulation) | ☐ 0 ☐ 1 |
| 3. | Peripheral sensitization (increased sensitivity at/near the tissue) | ☐ 0 ☐ 1 |
| 4. | Central sensitization (spinal/brain changes, amplification of pain) | ☐ 0 ☐ 1 |
| 5. | Descending modulation (inhibition/facilitation; role of the brain in “turning up/down” pain) | ☐ 0 ☐ 1 |
| 6. | Role of biopsychosocial factors (cognition, emotion, context, environment) in pain modulation | ☐ 0 ☐ 1 |
| 7. | Use of metaphors, drawings, or analogies to reconceptualize pain | ☐ 0 ☐ 1 |
| 8. | Brief case scenario(s) illustrating reconceptualization and biopsychosocial perspective | ☐ 0 ☐ 1 |

**PNE content adherence calculation (for PNE sessions only):**

- Total items delivered as planned = **(PNE1–PNE8 “1”s)** = ____ / 8
- **% Planned content delivered** = ____ %

**Meets adherence criterion** (≥90% of planned content delivered)?
☐ Yes  ☐ No

**B. If this is a Red-flags session (leave blank for PNE sessions)**

| **RF Item** | **Planned Red-Flags Content Component** | **Delivered as Planned? (No = 0, Yes = 1)** |
| --- | --- | --- |
| 1. | Definition and purpose of red-flags in low back pain assessment | ☐ 0 ☐ 1 |
| 2. | Serious pathology categories (e.g. malignancy, infection, fracture, cauda equina syndrome) | ☐ 0 ☐ 1 |
| 3. | Key history-taking features and systemic signs for serious pathology | ☐ 0 ☐ 1 |
| 4. | Indications for referral / further medical investigation | ☐ 0 ☐ 1 |
| 5. | Emphasis that session focuses on screening, not detailed pain neurophysiology | ☐ 0 ☐ 1 |

**Red-flags content adherence calculation (for Red-flags sessions only):**

- Total items delivered as planned = **(RF1–RF5 “1”s)** = ____ / 5
- **% Planned content delivered** = ____ %

**Meets adherence criterion** (≥90% of planned content delivered)?
☐ Yes  ☐ No

**SECTION 2 – Cross-Condition Contamination Check**

*(“no inclusion of materials from the other condition”)*

**Instruction:**
Record whether any content clearly belonging to the *other* condition was introduced.

A. **For PNE session only**

| **Item** | **Contamination Indicator (Content from Red-Flags Session)** | **Yes/No** |
| --- | --- | --- |
| 1. | Detailed teaching of red-flags lists (malignancy, infection, fracture, CES) as a central focus | ☐ Yes ☐ No |
| 2. | Prolonged discussion of referral criteria for serious spinal pathology (beyond brief mention) | ☐ Yes ☐ No |
| 3. | Session emphasis shifted from reconceptualizing pain to red-flag screening as primary learning aim | ☐ Yes ☐ No |

B. **For Red-flags session only**

| **Item** | **Contamination Indicator (Content from PNE Session)** | **Yes/No** |
| --- | --- | --- |
| 1. | Explanation of nociceptive transmission and neurophysiological pain mechanisms | ☐ Yes ☐ No |
| 2. | Discussion of central or peripheral sensitization | ☐ Yes ☐ No |
| 3. | Explicit framing of pain as a “neurophysiological output modulated by biopsychosocial factors” | ☐ Yes ☐ No |
| 4. | Use of PNE metaphors/diagrams (e.g. alarm systems, volume knobs, pain as “a protective output”) | ☐ Yes ☐ No |

**Contamination criterion:**

- **Meets criterion** if *all* contamination items for that session type are marked **No**.
- If any item is marked **Yes**, briefly describe in comments and flag as deviation.

**SECTION 3 – Overall Fidelity Rating for This Session**

1. **Planned content delivered ≥90%?**
   ☐ Yes  ☐ No
2. **No cross-condition content included?**
   ☐ Yes  ☐ No

**Overall session fidelity (as per protocol definition):**

- ☐ **Adherent** (Both criteria satisfied)
- ☐ **Non-adherent** (One or both criteria not satisfied)

Observer signature: _____________________  Date: ____ / ____ / ______

**Appendix S2.**

**Red-Flags Knowledge Test (RF-KT): Student Version**

**Instructions
The following questions relate to “red flags” in individuals presenting with low back pain. Red flags are features that may indicate serious underlying spinal pathology. Please answer each item by selecting Yes or No according to what you believe is correct. Select only one answer for each item.**

1. New urinary retention or overflow incontinence with back pain indicates possible cauda equina syndrome requiring urgent evaluation.
☐ Yes  ☐ No

2. Any single red flag automatically mandates immediate emergency referral.
☐ Yes  ☐ No

3. Unexplained weight loss with new low back pain warrants further evaluation for malignancy.
☐ Yes  ☐ No

4. Severe pain intensity alone is a reliable indicator of serious spinal pathology.
☐ Yes  ☐ No

5. A history of malignancy increases the likelihood that new back pain represents metastatic disease.
☐ Yes  ☐ No

6. New saddle anesthesia (numbness around the perineal/genital region) is a classic red flag for cauda equina syndrome.
☐ Yes  ☐ No

7. Stress incontinence (leakage with coughing or sneezing) is specific for cauda equina syndrome.
☐ Yes  ☐ No

8. Fever with spinal pain in a person with intravenous drug use or immunosuppression raises concern for spinal infection.
☐ Yes  ☐ No

9. Minor trauma in an older adult or someone with osteoporosis may be a red flag for vertebral fracture.
☐ Yes  ☐ No

10. Multiple red flags always prove a sinister cause.
☐ Yes  ☐ No
